# A scoping review on HIV early infant diagnosis among HIV exposed infants, ART use and adherence in Tanzania

**DOI:** 10.1186/s12879-023-08868-8

**Published:** 2023-12-11

**Authors:** Emmy Metta, Novatus Tesha

**Affiliations:** 1 Department of Behavioural Sciences, School of Public Health and Social Sciences, Muhimbili University of Health and Allied Sciences, MUHAS, P.O. Box 65015, Dar es Salaam, Tanzania; 2 Department of Development Studies, School of Public Health and Social Sciences, Muhimbili University of Health and Allied Sciences, MUHAS, P.O. Box 65015, Dar es Salaam, Tanzania

**Keywords:** Infants, HIV exposed, HIV diagnosis, HEID uptake, Loss to follow-up, Adherence, Tanzania, Scoping review

## Abstract

**Background:**

HIV Early Infant Diagnosis (HEID) is critical for the timely initiation of HIV treatments and improved health outcomes among HIV-infected infants. However, the uptake of HEID remains largely low in Tanzania. This descriptive scoping review of evidence on HEID among HIV-exposed infants in Tanzania seeks to understand the dynamics of HEID, ART use and adherence to inform targeted interventions and promote its uptake.

**Methods:**

The Arksey and O’Malley’s methodological framework guided this scoping review. We searched for peer-published articles on HEID in Tanzania through PubMed and Google Scholar with full-text retrieval from HINAR. We included only English language articles published between 2013 and 2022. Further searches for the materials on the reference lists of the publications yielded additional relevant articles. We carried out an inductive thematic analysis to analyze and synthesize the data.

**Results:**

In all, nine articles met the inclusion criteria and, hence, qualified for the review. The variations in the uptake of HEID in the empirical literature reviewed indicate an upward trend. HEID increased from 77.2% in 2009 to 97.8% in 2011 in Kilimanjaro, Mbeya and Tanga and from 53.2% in 2014 to 69.2% in 2016 in Dar es Salaam. The median age at the initial test ranged from 5.6 weeks in Kilimanjaro to 8.6 weeks in Mbeya. The uptake of HEID was necessitated by individual, household and health facility factors. Unknown HIV status at conception, low level of education of the household head, and large household size negatively affected uptake of HEID. The health facility factors such as unavailability of the test kits, long distances to the facility and transport costs negatively influenced HEID uptake. The percentage of HIV-positive infants on ART ranged from 52.7 to 61%, and loss to follow ranged from 31 to 61%.

**Conclusion:**

The uptake of HEID varied among regions depending on individual, household and health facility factors. Multifaced efforts are needed to address these factors for accelerated uptake of HEID and improved health outcomes and survival among this strategic population group.

**Supplementary Information:**

The online version contains supplementary material available at 10.1186/s12879-023-08868-8.

## Background

Children aged 0–14 years account for 5% of all the people living with HIV, 10% of the new HIV infections and 15% of all AIDS-related deaths globally [1]. Under-two HIV-positive infants have a higher risk of disease progression and mortality than older children [2] and non-exposed ones [3]. In fact, about a third of the infected infants die before their first birthday and more than half of the remaining die before their second birthday [4]. Such otherwise preventable early deaths are unacceptable since the lives of these infants could be elongated with early and timely diagnosis and treatment [5]. The World Health Organisation (WHO) recommends HIV Early Infant Diagnosis (HEID) and treatment for HIV-exposed infants as a key strategy for achieving the 2030 global HIV elimination goal. Indeed, HEID provides an opportunity for early identification of the HIV-infected children for timely initiation of the antiretroviral treatment aimed to improve child health outcomes [6]. Yet, only 51% of the HIV-exposed infants receive an HEID at week 6 of age and it is less than half of those tested that are initiated on ART [7].

Similar to most Sub-Saharan Africa (SSA) countries, paediatric HIV is of grave concern in Tanzania. About 11% of all the HIV infection in the country is acquired through Mother-to-Child Transmission (MTCT) [1] during pregnancy, at childbirth, and/or through breastfeeding [8]. Early initiation of antiretroviral therapy (ART) to HIV exposed infants lowers the risks of serious clinical conditions, forestalls the development of full blown AIDS, and progression to death, thus increasing their survival chances [9, 10]. However, initiating ART for HIV-infected infants can only be possible only after establishing their HIV status [11].

HEID in Tanzania is part of the maternal and child health services package that has been integrated into the Prevention of Mother-to-Child Transmission of HIV (PMTCT) interventions. To improve access to HEID for exposed infants, the Tanzania government is implementing the WHO-recommended Option B + guidelines strive to foster early identification and initiation of treatment for HIV-infected infants regardless of the clinical signs of the disease. Under Option B+, all HIV-exposed infants receive an HIV early infant diagnosis test at 4–6 weeks and, then, at six months after being weaned from breastfeeding followed by another test at 18 months for final diagnosis [12].

Despite the high percentage (90%) of pregnant women enrolled in prevention of mother-to-child transmission of HIV services the uptake of HIV early infant diagnosis is reportedly low in the country [13]. Thus, it is imperative to establish the dynamics of HEID, ART use and adherence is important towards informing targeted interventions aimed to promote its uptake and policy evaluation to accelerate reaching the national and international targets of “ending AIDS as a public health threat by 2030”. This study reviewed published information on HEID, ART use and adherence among HIV-exposed infants in Tanzania covering the 2013–2022 period to document the type and extent of information available among HIV-exposed infants in Tanzania.

## Methods

The review followed the recommended Arksey and O’Malley’s five scoping review methodological framework stages: (i) Identifying the research question, (ii) Identifying relevant studies, (iii) Study selection, (iv) Charting the data, (v) Collating, summarising and reporting results [14]. Applying this framework, we wanted to answer questions, first, on what constitutes the existing evidence on HEID, ART use and adherence among HIV- exposed infants in Tanzania, and second, on the type and extent of information available on HEID in the country.

### Search strategy

We conducted a comprehensive literature search to identify original articles on HIV Early Infant Diagnosis in Tanzania. Searching for these documents focused on PubMed and Google Scholar using a set of comprehensive topics-related search terms. The full-text retrieval of the identified articles was done through HINARI. We included original articles written in English on original work conducted in Tanzania and published in reputable peer-reviewed journals using either qualitative or quantitative approaches and involving aged infants up to two years. We excluded working papers, systematic reviews, and articles including pregnant women newly-diagnosed with HIV or initiated on Option B + because of pregnancy or breastfeeding and articles on health care costs associated with clinic visits for prevention of mother-to-child transmission of HIV.

The search was restricted to articles published between 1st January 2013 and 31st December 2022. This time-span ensure that the articles retrieved reflected the current guidelines on paediatric HIV diagnosis and management in Tanzania. In addition, background citation tracking on the eligible studies was applied to identify additional sources of information.

The search strategy included a range of relevant combinations of keywords that were combined using the Boolean operators ‘AND’ to narrow the search appropriately and ‘OR’ to expand it with similar terms. The following strings were included in the search strategy: ‘infant OR ‘children’ OR paediatric AND HIV infection OR AIDS AND Tanzania; infant” OR ‘children’ OR ‘paediatric’ AND HIV diagnosis AND Tanzania; infant” OR ‘HIV exposed infant’ OR “HIV exposed children’ OR ‘HIV-exposed infant’ OR “HIV-exposed children’ OR ‘paediatric’ OR ‘early’ OR ‘new-born’ AND HIV diagnosis AND Tanzania; infant” OR ‘children’ OR ‘paediatric’ OR ‘early’ AND HIV testing AND Tanzania; ‘infant’ OR ‘children’ OR ‘paediatric’ AND ART use AND Tanzania; ‘infant’ OR ‘children’ OR ‘paediatric’ AND loss to follow up AND Tanzania; ‘infant’ OR ‘children’ OR ‘paediatric’ AND ART adherence AND Tanzania; ‘infant’ OR ‘children’ OR ‘paediatric’ AND retention to ART services AND Tanzania. The article inclusion was based on the following criteria: Original studies conducted in Tanzania, used quantitative or qualitative or both approaches, covered HIV-exposed infants, published in English, and conducted between 2013 and 2022.

retention to ART services.

**Fig. 1:**
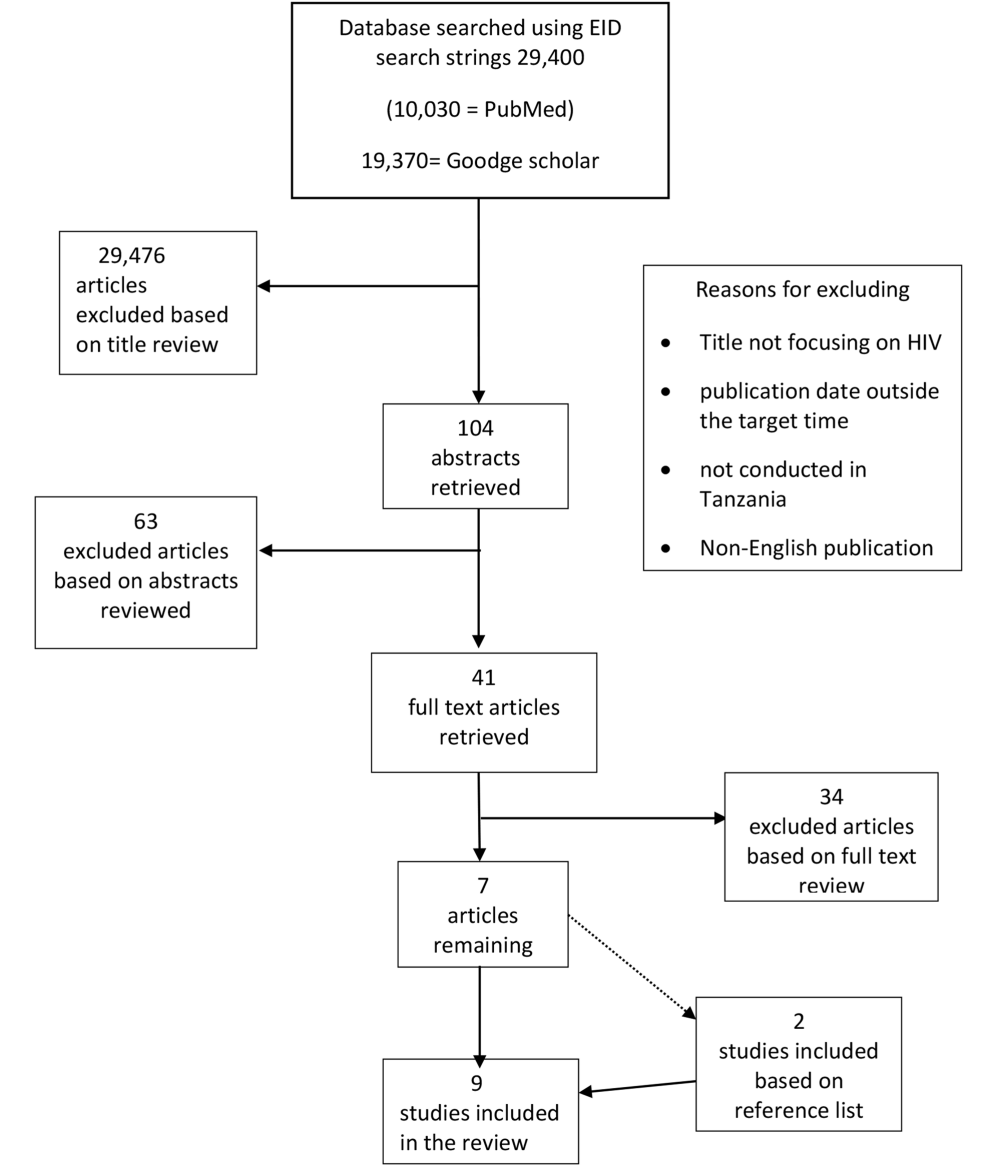
Process of selecting articles

The search resulted in 29,400 articles as depicted in Fig. 1. Twenty-nine thousand, four hundred and seventy-six of these were excluded for reasons including the title not focusing on HIV diagnosis, language other than English, or publication date outside targeted time-frame. For the 104 titles we found to be relevant to our subject area, we retrieved their abstracts to determine whether they matched our criteria. Following this screening exercise, we excluded 63 abstracts based on content relevance to our study focus. The remaining 41 abstracts met the set criteria. As such, their full texts were retrieved from HINARI for further scrutiny. The review of these full texts excluded 34 articles due to their focus on population. Finally, only 7 articles remained to match our inclusion criteria. References of the documents retrieved were subjected to further searches to access more relevant and related papers. This search yielded two more articles.

### Charting the data

Each publication was thoroughly reviewed by each author independently by sifting, charting and sorting the information into a data charting form, a structured data sheet designed specifically for that purpose. The information extracted from each reviewed study were as follows: (i) Author, (ii) title, (iii) publication year, (iv) study site, (v) study design, (vi) study population, (vii) participant age, (viii) study design, (ix) data collection methods, (x) sample size, (xi) HIV testing among exposed infants, (xii) determinants of HIV testing, (xiii) HIV exposed infants on ART, and (xiv) loss to follow-up. The independent review was followed by discussions among the authors on the content of the results and where disagreements prevailed resolutions were reached by consensus.

### Collating, summarising, and reporting the results

A narrative account of the data extracted from the included studies was analysed using thematic content analysis based on the themes identified inductively. The inductive themes extracted were based on the research objective. An inductive thematic analysis was done with the support of framework method for analysis [14]. Data were extracted around the following outcomes: Proportion of HIV testing among HIV exposed infants; age at HIV testing among exposed infants and factors associated with the uptake HEID; HIV-positive infants on ART, loss to follow-up and mortality among HIV-positive infants.

### Findings

A total of nine studies were reviewed and all were quantitative studies. The sample size in the studies ranged from 383 to 29,531. The reviewed studies were conducted in Mbeya [2], Mwanza [1], Iringa [1], Tanga [1] and Dar es Salaam [1]. There were also two study with national coverage, one done in three regions (Tanga, Mbeya and Kilimanjaro). These studies were published between 2013 and 2022, mostly from 2018 to 2022 see annex 1. The included studies applied different research designs: cross-sectional, descriptive-analytical, retrospective, prospective diagnostic cohort, and interventional study.

### Proportion of HIV testing among HIV exposed infants

The proportion of HIV testing among HIV-exposed infant was reported by five studies. These studies were carried out in Kilimanjaro, Mbeya, Tanga, Dar es Salaam, Iringa, Tanga, and a national level study [6, 15–18]. At the national level, the proportion of HEID increased from 92.7% in 2017 to 96.4% in 2019 on the 1st test whereas test 2 and 3 recorded lower uptake [18]. In Kilimanjaro, Mbeya and Tanga the proportion of HIV testing among exposed infants increased from 77.2% in 2009 to 97.8% in 2011 [15]. In 2018, it was reported to be 75.6% in Mbeya. Moreover, there was increase from 53.2% in 2014 to 69.2% in 2016 in Dar es Salaam [16]. For the studies conducted in Muheza district in Tanga Region and Iringa region the proportion of HIV-exposed infants after testing was 57.1% and 34.6%, respectively [6, 17].

### Age at HIV testing among exposed infants

Seven studies reported on the age of HIV testing among HIV-exposed Infants (HEI). These studies were carried out in Mbeya, Tanga, Mwanza, Kimanjaro and Iringa regions. The studies reviewed show that 75.6% of the infants received HIV tests between the recommended 8 weeks in Mbeya and 59.1% in Dar es Salaam [16, 19]. In Muheza district, Tanga region, 57.1% of the infants accessed the first HIV test between 4 and 6 weeks of age with the median age of 6 weeks (IQR: 6–20 weeks) [6]. The median age of testing (2rd PCR) at six weeks after cessation was 14 months (ranging from 13.5 to 18 months) in Iringa with a proportion of those who were tested accounting for 34.6% [17]. The median age of infants at their first test at 8.57 weeks in Mbeya, 7.95 weeks in Tanga, and 5.6 weeks in Kilimanjaro [15]. The mean age at initial test was 16.5 weeks (range 4–99 weeks) in the Mwanza study [20].

### Factors associated with uptake of HIV testing among HIV exposed infants

Three studies reported on the factors associated with the early uptake of HIV testing among HIV-exposed infant [6, 16, 17]. Individual, household and health facility factors emerged to be associated with uptake of HIV testing in the studies. The individual factors for low-uptake of HEID were unknown HIV status at the conception, HEI born to mothers with WHO stage II, stage III, and stage IV [16, 21]. Mothers with adequate knowledge on prevention of mother-to-child transmission (PMTCT), awareness of HIV testing before or during pregnancy and being attached to psycho-social support groups had higher chances for the uptake of HEID [17]. Household factors such as those with a household head with at least a high education level had higher chances of accessing HEID than those with a low education level and large family size [6]. The health facility factors observed for low uptake of HEID were unavailability of the test kits, long distance to the facility and transport costs [17].

### HIV-positive infant on ART

HIV infants on ART was reported in three studies [19, 22, 23]. At the national level, the proportion of infants on ART was observed to increase from 61% in 2017 to 90% in 2022 [22, 23]. In Mbeya region, only 52.7% of HIV-positive infants aged less than two years were on ART during 2014–2016 period [19]. This figure was lower than 61% that was reported for HIV = positive children aged 0–14 years who were on ART between 2011 and 2014 in the country [23].

### Loss to follow-up

Only two studies reported information on loss to follow-up of infants on ART [15, 23]. There is a substantial loss to follow-up of children and infants at all stages of HEID. The overall results in the studies conducted in Kilimanjaro, Mbeya and Tanga indicated 61% lost to follow-up during 2009 and 2011 [15]. The other study that had a national coverage reported 31% lost to follow-up between 2011 and 2014 in the country [23].

## Discussion

The studies reviewed indicated an increasing trend of HIV testing at the age of less than 8 weeks among exposed infants in Dar es Salaam, Kilimanjaro, Mbeya and Tanga. This could be a result of the expansion of the PMTCT programme services provision that has also been reported in other settings as effective in increasing HIV testing among infants [24]. PMTCT services are provided to more than 91% of the Reproductive, Child Health clinics in Tanzania [25]. Despite the increasing trend, the percentage of infants who accessed the EID within the recommended age was still below the national target of 95% [25]. There was variation across regions: 75.6% in Mbeya, 59.1% in Dar es Salaam, 57.1% in Muheza Tanga and 34.6% in Iringa for the test done six weeks after cessation of breastfeeding. With the exception of Mbeya, the prevalence in the other regions was found to be below the national average of 66% [1]. This difference was associated with lack of testing laboratories, stock out of testing kits, low coverage of HEID services and remoteness of some areas [15]. There is also a need for studies to assess regional variations in HEID and associated factors to inform context-specific programmes for improved uptake.

The studies reviewed indicated variations in the median age at the initial HEID test ranging from 5.6 to 8.6 weeks with differences across areas. These results are consistent with those reported in other studies conducted in Sub-Saharan Africa including in Uganda that reported a median age of 5.4 weeks, Mozambique 5 weeks, and Zambia 8.1 weeks and India 8 weeks [26–29]. HEID is an initial step before initiating early infant antiretroviral therapy to halt chances for rapid disease progression and mortality among the infected infants [30]. There is also a need for concerted efforts aimed to boost the demand for HEID among mothers living with HIV to increase the chances of enjoying likely survival benefits associated with early HEID performance.

Several factors emerged to be contributing to low uptake of HEIDs in the studies reviewed. These included individual factors such as mother unknown HIV status at conception, inadequate knowledge on prevention of mother-to-child transmission of HIV services; household factors such as low level of education of the head of household, large household size, non-attachment to psycho-social support groups, limited household support and/or permission to undergo the test; and health facility factors such as long distances to the health facilities and transport costs, and unavailability of test-kits. This review results collaborates with what was reported in other Sub-Saharan Africa settings and elsewhere [31–33]. Low uptake of HEID also contributes to stressful situations, particularly because through HEID the health status of the HIV-exposed babies will be known to inform early linkage to care and treatment for improved health outcomes. Yet, none of the studies reviewed included information on the underlying mechanisms shaping the mothers’ decision-making process on the uptake of HEID. Such information is crucial not only for informing targeted interventions but also for value-adding policy decision to facilitate early diagnosis of HIV among exposed infants.

Retention of HIV-infected infants on ART is one of the key themes that emerged in the review. The proportion of HIV infant on ART was reported at 61% in the nation-wide study with the other study reporting 52.7% for Mbeya region [19, 23] This proportion is lagging the national and global target of 95% for all people living with HIV/AIDs enrolling in ART [25]. The lower level of HIV-infected infants in Tanzania on ART is a shared concern in many other countries where even lower percentage are reported such as 22% in Senegal, 37% in Uganda, 38% in Cambodia and less than 50% in Malawi [24, 34].

This low percentage on ART treatments could be explained by the higher rates of loss to follow-up. In the studies reviewed loss to fallow-up was reported at 61% for the study done in Kilimanjaro, Tanga, and Mbeya and 31% at the national level [15, 23]. Similar findings have also been reported from outside Tanzania where substantial percentages are reported for loss to follow-up on HIV-exposed infants: 48% in Malawi, 50% in Central Mozambique, 67% in Nigeria and 43% in Uganda 43% [34–36]. The reasons for non-ART adherence and loss to follow-up could be attributable to stigma, discrimination, lack of knowledge on PMTC, and limited accessibility of health facilities; however, none of the studies reviewed detailed these aspects [37]. As such, there is a need to explore and gain detailed insights into the contextual issues that can inform paediatric non-ART adherence and loss to follow-up. In this regard, conducting qualitative studies to uncover the underlying mechanisms shaping the uptake of HEID and adherence to the ART in this population group could help to underpin and inform future interventions aimed to address this problem.

### Strength and limitations of the review

The strength of this review is the engagement of a comprehensive search terms of relevant studies. The review used a systematic approach to identifying relevant studies, charting, data extraction and analysis. Even though the inclusion of only English language published articles might have introduced selection bias and limit the retrieval of, for example, in KiSwahili, the national language published articles, there is no KiSwahili language research journal from which we could have accessed such articles. We are, therefore, confident that inclusion of English only articles did not translate into missing some salient information. Nevertheless, this scoping review provides a holistic overview of the evidence available on HEID among HIV-exposed infants in Tanzania, which may contribute to the sharping of the development of interventions aimed to improve health outcomes and reaching the national targets of ending AIDS as a public health threat by 2030.

## Conclusion

The empirical evidence shows that HEID, ART use and adherence is a challenge in Tanzania shaped by low uptake and delayed HIV diagnosis as well as loss to follow-up and retention to ART services. Aspects surrounding individual, household and health facility emerged to be the triggering factors. As such, multifaced efforts embracing all these factors—individual, household and health facility—are needed to strengthen both the demand for and uptake of HEID services for improved health outcomes and survival among this strategic population group.

### Electronic supplementary material

Below is the link to the electronic supplementary material.

Supplementary Material 1

## Data Availability

All the data generated and analysed during this study are included in the article (Table S1).
