# Supplementary material for: A scoping review on HIV early infant diagnosis among HIV exposed infants, ART use and adherence in Tanzania
Source: BMC Infect Dis. 2023 Dec 11;23:868. doi: 10.1186/s12879-023-08868-8 (PMC10714633; doi:10.1186/s12879-023-08868-8)
Supplement: Supplementary file 1 — Supplementary Material 1 [file 12879_2023_8868_MOESM1_ESM.doc]

**Table S1: Summary of characteristics of the articles included in the scoping review**

| **S/N** | **Author/year of publication** | **Study design** | **Geographical location** | **Sample size** | **Study title** | **Population** | **Summary of findings** |
| --- | --- | --- | --- | --- | --- | --- | --- |
| 1. | Mercy G Chiduo et al, 2013 | Cross sectional | Kilimanjaro, Mbeya and Tanga | 4,860 | Early infant diagnosis of HIV in three regions in Tanzania; Successes and challenges. | HIV Exposed Infants | Overall proportion of tested infants in the in Kilimanjaro, Mbeya and Tanga regions increased from 77.2% in 2009 to 97.8% in 2011.  Median age of infant at first test was 8.57 weeks in Mbeya, 7.95 in Tanga, and 5.6 weeks in Kilimanjaro.  HIV prevalence among HIV Exposed Infants was 10.5% |
| 2. | Michael F. Mboya et al, 2020 | Cross sectional | Dar es Salaam | 12,117 | Trend and predictors for early infant diagnosis by PCR among HIV-exposed infants in Dar es Salaam region , Tanzania | HIV-infected mothers and their exposed infants up to 18 months | The proportion of HIV exposed infant who were tested for HIV by age of 8 weeks increased from 53.2% in 2014 to 69.2% in 2016.  The median at age at testing was 1.4 months (1.7 – 3.1)  Factors for high uptake of EID were replacement feeding, receiving nevirapine, and prophylaxis, while for low uptake was mothers WHO HIV stage of disease progression II, III, and IV.  HIV prevalence 2.3% among HEI |
| 3. | Veneranda M. Bwana et al 2018 | Cross sectional | Muheza- Tanga | 836 | Accessibility of Early Infant Diagnostic Services by Under-5 Years and HIV Exposed Children in Muheza District, North-East Tanzania | Mother/guardian of under 5 children born to HIV positive mother who were not breastfeeding for >= 6 weeks. | 57.1% of children accessed EID between 4 and 6 weeks of age.  The median age at the first HIV test was 6 weeks (IQR: 6-20 Weeks)  Predictors of missed HEID were unknown HIV status at conception (AOR = 0.6, 95% CI 0.4–0.8) and infants with ages 13–59 months (AOR = 0.4, 95% CI 0.2–0.6).  Children living with a head of household with at least a high education level had higher chances of accessing EID (AOR = 1.8, 95% CI 1.1–3.3). Children’s chances of accessing EID services was three-fold higher among mothers/guardians with good knowledge of HIV infection prevention of mother-to-child transmission (AOR = 3.2, 95% CI 2.0–5.2) than those with poor knowledge.  The prevalence of HIV was 10.6 % |
| 4. | Sophia Samson et al, 2018) | Cross sectional | Iringa | 414 | Uptake of early infant diagnosis (EID) at six weeks after cessation of breastfeeding among HIV exposed children: A cross sectional survey at six high volume health facilities in Iringa, Tanzania | HIV exposed infants aged between 13.5 and 18 months | 34.6% of HIV exposed children received the HIV test six week after cessation of breastfeeding.  Median Age at testing was 14 Months (Range between 13.5 – 18 Months)  Factors for high uptake of EID were adequate knowledge on PMTCT, living in urban areas, awareness of the test before pregnancy or during pregnancy and being attached to psychosocial support groups.  Health-system-related factors for missed EID were unavailability of the test kits and long distance from home. |
| 5. | E. Mgelea et al, 2016 | Retrospective analysis | Mwanza | 10,454 | HIV Positivity rate and long turnaround time of early infant diagnosis of HIV infection testing results in Lake Zone, Tanzania | HIV exposed infants | The mean age at initial EID was 16.5 weeks (Range 4 -99 weeks)  HIV prevalence among the HEI was 8.5% |
| 6. | Somi et al, 2017 | Descriptive Analytical | National wide study | 29, 531 | Pediatric HIV care and treatment services in Tanzania: Implications for survival | HIV positive children aged 0 – 14 years | 31% were Lost to Follow-up  61% were on care or ART  8% died and the probability of death was 31%, 43% 52% and 61% by 1, 2, 5 and 10 years of age, respectively. The hazard of death was greatest at very young ages. |
| 7. | O. Mwashiuya et al, 2018 | Cross sectional study | Mbeya | 1,176 | Implementation of early infant diagnosis of HIV in Mbeya region, Tanzania | HIV exposed infants between 0 – 2 years | 75.6% received HIV test between the 8 recommended weeks.  HIV prevalence was 4.7%  52.7% of those who were HIV positive were initiated on antiretroviral therapy |
| 8 | Mangu Changa et al., 2022 | Retrospective study | Tanzania |  | HIV Early Infant Diagnosis (HEID) uptake and outcomes among HIV exposed infants in Tanzania: a retrospective study using national CTC2 dataset. | HIV exposed infants | There was an increase in the initial HEID uptake from 92.7% in 2017 to 96.4% in 2019. However, there was low uptake of test 2 and test 3 with the declining retention of mother-child pairs from 85.6% at week 6 to 71.1% at month 18. However, 49% of all cases detected by month 18 resulted from only 70% of retained infants. An increase in nevirapine uptake associated with declining MTCT positivity was noted. |
| 9. | Olomi, Willyhelmina et. Al 2022 | Prospective study | Tanzania | 640 children/629 mothers | HEID and uptake of postnatal prophylaxis among infants born to HIV positive mothers: a prospective study | HIV mother-infant pair | The positivity rate at 6 weeks post-delivery was 0.3% (2 HIV-infected babies out of 640) with no incremental yield in positivity when an additional PoC HEID test was repeated at week 12 post-delivery. More than 90% of the HIV-exposed infants were initiated on Nevirapine mono prophylaxis, while 8/96 (8.3%) were categorized as high-risk for Mother to Child Transmission (MTCT) and were initiated on enhanced prophylaxis. In those who were categorized as high risk, only 6/96 (6.3%) had nucleic-acid test at birth as per the Tanzanian National HIV testing algorithm. |
